# Cleavage of Hsp70.1 causes lysosomal cell death under stress conditions

**DOI:** 10.3389/fmolb.2024.1378656

**Published:** 2024-05-27

**Authors:** Tetsumori Yamashima, Daria Mochly-Rosen, Soichi Wakatsuki, Eishiro Mizukoshi, Takuya Seike, Isabel Maria Larus, Che-Hong Chen, Miho Takemura, Hisashi Saito, Akihiro Ohashi

**Affiliations:** ^1^ Department of Psychiatry and Behavioral Science, Kanazawa University Graduate School of Medical Sciences, Kanazawa, Japan; ^2^ Department of Gastroenterology, Kanazawa University Graduate School of Medical Sciences, Kanazawa, Japan; ^3^ Department of Chemical and Systems Biology, Stanford University School of Medicine, Stanford, CA, United States; ^4^ Department of Structural Biology, Stanford University School of Medicine, Stanford, CA, United States; ^5^ Laboratory of Gene Function, Research Institute for Bioresources and Biotechnology, Ishikawa Prefectural University, Nonoichi, Japan; ^6^ Division of Collaborative Research and Development, Exploratory Oncology Research and Clinical Trial Center, National Cancer Center, Kashiwa, Japan

**Keywords:** ALDH2, calpain–cathepsin hypothesis, chaperone-mediated autophagy, hydroxynonenal, Hsp70.1, LAMP2A, lysosomal cell death, lifestyle-related disease

## Abstract

Autophagy mediates the degradation of intracellular macromolecules and organelles within lysosomes. There are three types of autophagy: macroautophagy, microautophagy, and chaperone-mediated autophagy. Heat shock protein 70.1 (Hsp70.1) exhibits dual functions as a chaperone protein and a lysosomal membrane stabilizer. Since chaperone-mediated autophagy participates in the recycling of ∼30% cytosolic proteins, its disorder causes cell susceptibility to stress conditions. Cargo proteins destined for degradation such as amyloid precursor protein and tau protein are trafficked by Hsp70.1 from the cytosol into lysosomes. Hsp70.1 is composed of an N-terminal nucleotide-binding domain (NBD) and a C-terminal domain that binds to cargo proteins, termed the substrate-binding domain (SBD). The NBD and SBD are connected by the interdomain linker L_L1_, which modulates the allosteric structure of Hsp70.1 in response to ADP/ATP binding. After the passage of the Hsp70.1–cargo complex through the lysosomal limiting membrane, high-affinity binding of the positive-charged SBD with negative-charged bis(monoacylglycero)phosphate (BMP) at the internal vesicular membranes activates acid sphingomyelinase to generate ceramide for stabilizing lysosomal membranes. As the integrity of the lysosomal limiting membrane is critical to ensure cargo protein degradation within the acidic lumen, the disintegration of the lysosomal limiting membrane is lethal to cells. After the intake of high-fat diets, however, β-oxidation of fatty acids in the mitochondria generates reactive oxygen species, which enhance the oxidation of membrane linoleic acids to produce 4-hydroxy-2-nonenal (4-HNE). In addition, 4-HNE is produced during the heating of linoleic acid-rich vegetable oils and incorporated into the body via deep-fried foods. This endogenous and exogenous 4-HNE synergically causes an increase in its serum and organ levels to induce carbonylation of Hsp70.1 at Arg469, which facilitates its conformational change and access of activated μ-calpain to L_L1_. Therefore, the cleavage of Hsp70.1 occurs prior to its influx into the lysosomal lumen, which leads to lysosomal membrane permeabilization/rupture. The resultant leakage of cathepsins is responsible for lysosomal cell death, which would be one of the causative factors of lifestyle-related diseases.

## Background

**FIGURE 1:**
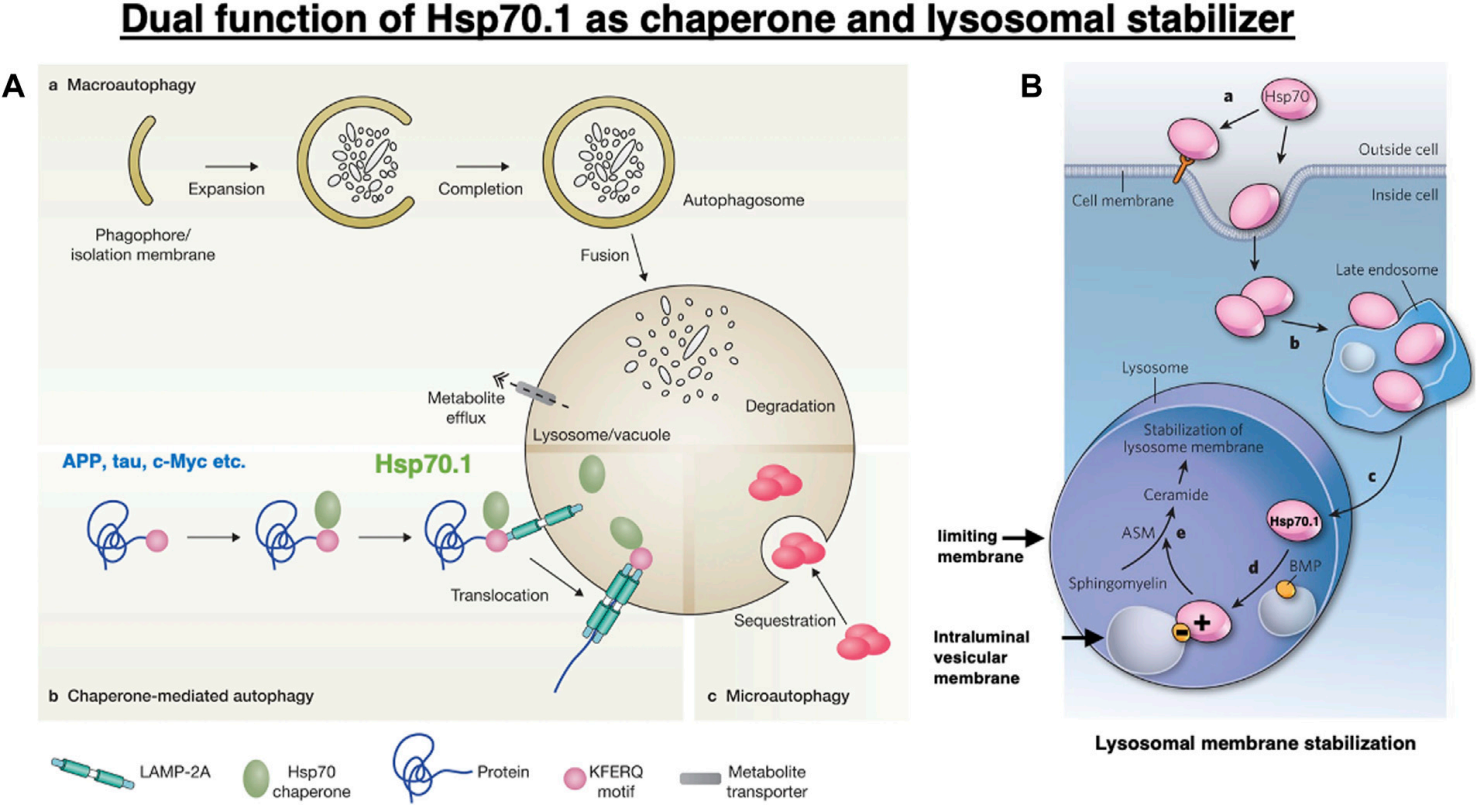
Dual functions of heat shock protein 70.1 (Hsp70.1) as a chaperone protein **(A)** and a lysosomal membrane stabilizer **(B)**. **(A)** Among the three pathways of autophagy, Hsp70.1 is indispensable for chaperone-mediated autophagy (b) in birds and animals. Under stress conditions, the cargo proteins with the Lys-Phe-Glu-Arg-Gln (KFERQ) motif, such as amyloid precursor protein (APP), tau protein, and c-Myc, are trafficked from the cytosol into the lysosomal lumen. Reprinted with permission from Boya et al. (2013). **(B)** Within lysosomes, the binding of positively charged Hsp70.1 with negatively charged bis(monoacylglycero)phosphate (BMP) facilitates the activation of acid sphingomyelinase (ASM) and the generation of ceramide, which contribute to the stabilization of lysosomal membranes. Adapted with permission from Horváth and Vígh (2010).

Heat shock proteins (Hsps) are molecular chaperones that can protect cells from physical and chemical hazards such as high temperature, hypoxia, free radicals, cytokines, ethanol, and chemical denaturants (Smith et al., 1998; Tsukahara et al., 2000; Balogi et al., 2019). When cells are subjected to such hazards, the synthesis of Hsps is immediately triggered, whereas that of most other proteins is arrested. Thus, Hsps can regulate cellular homeostasis and maintain cell survival (Rodríguez-Ariza, et al., 2005). Their major classes are grouped according to their molecular weight, which are as follows: small Hsp (Hsp10, Hsp25/27, and Hsp40), Hsp60, Hsp70, Hsp90, and Hsp100 (Horváth et al., 2008). Hsp70 is a representative stress-inducible protein that protects cells from various kinds of stress. It exhibits dual functions as a chaperone protein (Figure 1A) and a lysosomal membrane stabilizer (Figure 1B) (Kirkegaard et al., 2010; Balogi et al., 2019; Yamashima et al., 2020; Yamashima et al., 2023a; Yamashima, 2023b). Hsp70 restores the cell proteome by assisting in the refolding of denatured proteins and trafficking damaged/aged proteins from the cytosol into lysosomes, where they are recycled into amino acids. By interacting with lysosomal membrane lipids, Hsp70 also contributes to the stabilization of lysosomal membranes *via* generating ceramide from sphingomyelin (Figure 1B) (Horváth and Vígh, 2010; Kirkegaard et al., 2010; Balogi et al., 2019). This function of maintaining the integrity of the lysosomal limiting membrane is critical for cell survival since the lysosomal lumen is very acidic and contains hydrolases that are lethal to the cells if leaked.

The information about the number of human Hsp70 family members is still inconsistent (Brocchieri et al., 2008; Clerico et al., 2020; Nitika et al., 2020). The Hsp70 family shows high homology, sharing several functions in protein assembly, but the level of each chaperone protein in response to a stress condition may vary. There are two primary classes in the Hsp70 family: heat shock cognate protein 70.1 (Hsc70.1) and heat shock protein 70.1 (Hsp70.1). Although Hsc70.1 shares 85% amino acid similarity with Hsp70.1, Hsc70.1 and Hsp70.1 show structural differences in their carboxyl-terminal domain, which is related to the substrate specificity and particular biological functions (Ali et al., 2003; Ahn et al., 2005). Hsc70.1 is constitutively expressed for the chaperoning function under unstressed conditions and is induced mildly under stress conditions. In contrast, Hsp70.1 is expressed at very low levels under normal conditions, but it is highly induced under stress conditions, allowing cells to cope with acute negative factors affecting the proteome (Gebauer, et al., 1998; Chen et al., 2006; Liu et al., 2012).

Hsc70.1 is the constitutive form that is recruited by the cell as a primary defense against unfavorable conditions. In Alzheimer’s disease, for example, the expression of Hsc70.1 has been proposed as a defense mechanism in response to amyloid fibril formation by inhibiting the self-assembly of polyglutamine proteins into amyloid-like fibrils (Dworniczak and Mirault, 1987). Hsc70.1 is involved in the degradation of proteins with abnormal conformations by binding to a particular peptide region and labeling it for proteolysis (Kouchi et al., 1999). Hsc70.1 is involved in the structural maintenance of the proteasomes and conformational recognition of misfolded proteins by proteases. As Hsc70.1 contributes to delaying the progression of Alzheimer’s disease, a decreased level of Hsc70.1 expression and/or an increased level of its oxidation may be related to the neuropathological and biochemical abnormalities in the Alzheimer brain (Castegna et al., 2002). Hsc70.1 can form a stable complex with newly synthesized Hsp70.1 upon heat shock (Brown et al., 1993). Hsc70.1 and Hsp70.1 interact with each other, showing essentially similar functions and oxidative injury. Since Hsc70.1 helps the proteolysis at proteasomes, while Hsp70.1 helps the proteolysis mainly at lysosomes, Hsp70.1 should be more closely related to the occurrence of lysosomal cell death than Hsc70.1. In addition, under oxidative stress conditions, Hsc70.1 associates with immunogenic peptides less quantitatively than Hsp70.1, and the secondary structure of Hsc70.1 is less strikingly changed than that of Hsp70.1 (Callahan et al., 2002). Therefore, in this review, we focused on Hsp70.1 to elucidate the implication of its structural changes for lysosomal cell death.

Protein carbonylation is an irreversible post-translational modification induced by severe oxidative stress, but its consequences are poorly understood. Sultana et al. (2010) confirmed elevated levels of Hsp70.1 carbonyls in patients with mild cognitive impairment and early Alzheimer’s disease. Hsp70.1 carbonylation is also increased in pathological states such as cerebral ischemia (Oikawa et al., 2009; Yamashima and Oikawa, 2009), type 2 diabetes (Kavanagh et al., 2016; Boontem and Yamashima, 2021), and nonalcoholic steatohepatitis (Seike et al., 2022; Yamashima et al., 2023c). Although the pathogenesis of each disorder is multifactorial, and the causal relation remains poorly understood, it is widely accepted that reactive oxygen species (ROS) play a critical role in the occurrence of these diseases. Inside the lysosomes, chemically reactive metals such as iron, copper, zinc, and cobalt generate ROS through Fenton-type chemical reactions, and this can lead to the oxidation and destabilization of membrane lipids (Kurz et al., 2010; Kiselyov et al., 2011). Recently, we proposed that the lipid peroxidation product 4-hydroxy-2-nonenal (4-: fourth carbon; hydroxy: OH; -2-: two carbon double bonds; none: 9 carbon atoms; nal: aldehyde) (called 4-HNE hereafter) may expand lysosomal membrane injuries by facilitating the “calpain-mediated cleavage of the carbonylated Hsp70.1” (Oikawa et al., 2009; Yamashima and Oikawa, 2009; Sahara and Yamashima, 2010).

The lipid peroxidation product 4-HNE is generated from ingested ω-6 polyunsaturated fatty acid (PUFA)-rich vegetable oils and/or from the endogenous peroxidation of biomembrane lipids under oxidative stress conditions. These exogenous and endogenous 4-HNE may synergically oxidize Hsp70.1, which facilitates the calpain-mediated cleavage of the oxidized Hsp70.1 to induce lysosomal membrane rupture and cell death under stress conditions (Yamashima et al., 2020). To elucidate the molecular mechanism of the calpain-mediated cleavage of carbonylated Hsp70.1, it is important to understand the three-dimensional structural changes of Hsp70.1, especially under stress conditions. Herein, we discuss the molecular mechanism of the structural changes in Hsp70.1 under stress conditions and its implication for lysosomal cell death.

## Post-translational modification of Hsp70.1 under cell stress conditions

In healthy, unstressed cells, Hsp70.1 is expressed at low or undetectable levels (Gebauer, et al., 1998; Chen et al., 2006; Liu et al., 2012). However, under cell stress conditions, e.g., via free radicals, hypoxia, and acidosis, Hsp70.1 expression increases remarkably, and the cytosolic protein translocates to the nucleus, late endosomes, or lysosomes, and in cancer cells, it is also localized to the extracellular leaflet of the plasma membrane (Figure 1B) (Horváth and Vígh, 2010; Balogi et al., 2019). Although the endosomal, lysosomal, and extracellular pools of Hsp70.1 are interconnected in a dynamic fashion (Figure 1B), the mechanism by which Hsp70.1 crosses the endosomal–lysosomal membrane or plasma membrane is not well understood. Phosphatidylserine confers a negative charge to the cytosolic leaflet of the plasma membrane and the endosomal membrane, allowing the recruitment of positively charged proteins (Yeung et al., 2008; Armijo et al., 2014). Since Hsp70.1 has a cluster of positively charged Arg and Lys residues in the lid of the substrate-binding domain (SBD), an interaction between the lid and membranous phosphatidylserine enables the anchoring of the SBD or the SBD–cargo complex to cell membranes (Arispe et al., 2002; 2004; Morozova et al., 2016; Lamprecht et al., 2018). Noncytosolic localization, membrane crossing, and lipid interactions of Hsp70.1 are associated with its influx into endosomes and lysosomes and the lysosomal membrane integrity. These unique functions may be affected by Hsp70.1 modification and the lipid composition that either interacts with or can be modulated by Hsp70.1 (Balogi et al., 2019).

In the hippocampal CA1 neurons of Japanese macaque monkeys after transient global brain ischemia, excessive μ-calpain activation at the lysosomal membrane causes its rupture, leakage of cathepsin enzymes through the limiting membrane, and neuronal death (Yamashima et al., 1996). This cascade was formulated by the author’s group as the “*calpain–cathepsin hypothesis*” in 1998 (Yamashima et al., 1998). However, the substrate of activated μ-calpain had remained unknown for a decade (Yamashima, 2004). In 2009, through the proteomics analysis comparing normal and ischemic hippocampal CA1 tissues of monkeys, we discovered that the target molecule of activated μ-calpain is the carbonylated Hsp70.1 (Oikawa et al., 2009; Yamashima and Oikawa, 2009). Particularly after the carbonylation at Arg469, which is localized at the interface of the lid and SBD, Hsp70.1 becomes vulnerable to cleavage by the activated μ-calpain. In the monkey experimental paradigm showing delayed CA1 neuronal death on days 5–7 after ischemia, 4-HNE-induced carbonylation at Arg469 of the hippocampal Hsp70.1 increased by approximately 9-fold on day 3 and by approximately 4-fold on day 5, compared to the non-ischemic control Hsp70.1 (Oikawa et al., 2009).

Although calpain participates in many processes of the cell life under the physiological condition, its regulatory system becomes impaired due to age-dependent oxidative stress and brain hypoxia, leading to the pathogenesis of neuronal dysfunction, degeneration, and cell death, as observed in many neuropathological conditions, including Alzheimer’s disease (Mohaman et al., 2019). Calpain is also activated in cardiometabolic diseases and significantly contributes to the progression of associated complications such as atherosclerosis, steatosis, and obesity (Miyazaki, 2023). Calpain cleaves Hsp70.1 involved in the monkey hippocampal CA1 tissue *in vitro* following incubation with synthetic 4-HNE or hydrogen peroxide (Sahara and Yamashima, 2010). In addition, the *in vitro* oxidation of Hsp70.1 involved within other brain tissues of monkeys also facilitated its proteolysis by μ-calpain (Yamashima et al., 2014; Liang et al., 2016). Subsequently, under diverse pathological conditions of monkeys and humans, the same cascade of the μ-calpain-mediated cleavage of the carbonylated Hsp70.1 was demonstrated to occur *in vivo* in the pancreas and liver that were exposed to consecutive injections of 4-HNE (Boontem and Yamashima, 2021; Seike et al., 2022). Furthermore, 4-HNE activates β-cell μ-calpain via GPR109A (Boontem and Yamashima, 2021) or hepatocyte μ-calpain via GPR120 (Seike et al., 2022), which permeabilizes the lysosomal membrane and increases cytosolic cathepsins. In these experimental paradigms, the loss of the normal function of Hsp70.1 due to 4-HNE-induced carbonylation, followed by calpain-mediated cleavage, was demonstrated to cause lysosomal cell death not only in the brain but also in the liver and pancreas (Sahara and Yamashima, 2010; Yamashima et al., 2020).

The role of Hsp70.1 in lysosomal cell death was supported by both genetic and functional evidence. Deletion of the Hsp70.1 genes in mice impaired cardiac contractile function, altered calcium handling, and was associated with mild hypertrophy (Kim et al., 2006). Global gene knockouts of constitutively expressed Hsp70.1 isoform Hsp70.5, Hsp70.8, or Hsp70.9 were lethal, suggesting that these chaperones play a critical role in cellular physiology (Daugaard et al., 2005; Daugaard et al., 2007; Nitika et al., 2020). In contrast, Hsp70 induction in mice by valproic acid attenuated nitrosourea-induced photoreceptor cell death (Koriyama et al., 2014). Based on these rationales, we proposed a concept that calpain- and 4-HNE-induced Hsp70.1 disorder synergically causes cell death through lysosomal membrane rupture and/or permeabilization. This presumably contributes to the progression of lifestyle-related diseases such as Alzheimer’s disease, nonalcoholic steatohepatitis, and type 2 diabetes (Yamashima et al., 2022).

## Hsp70.1 and chaperone-mediated autophagy

**FIGURE 2:**
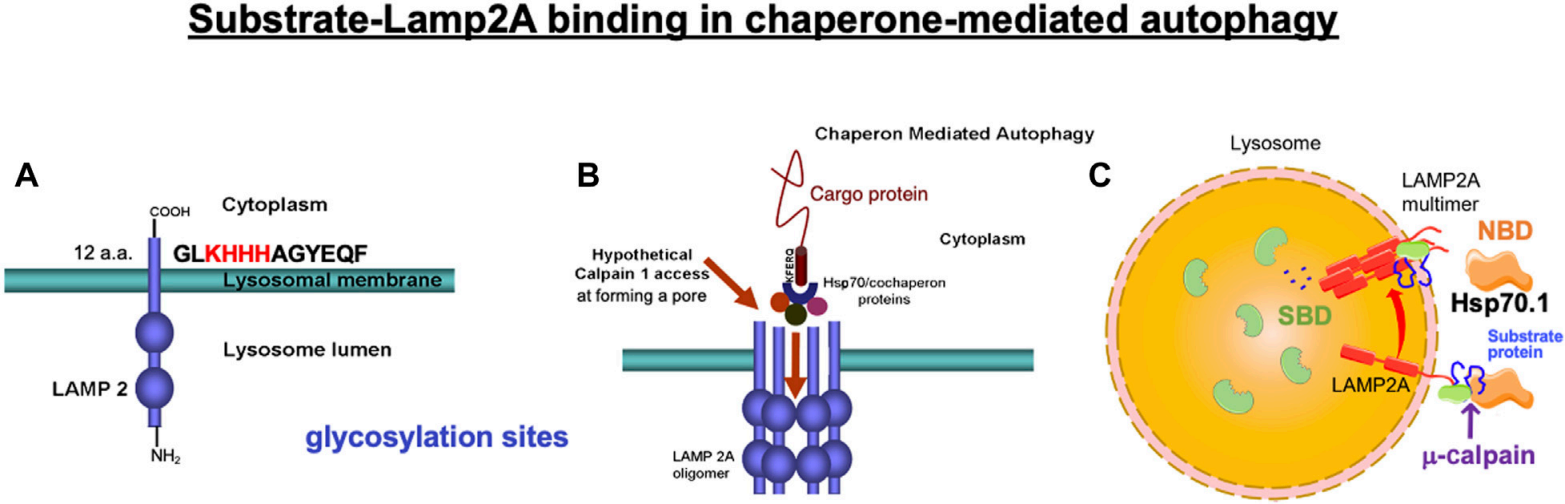
Binding of the cargo protein with lysosome-associated membrane protein type 2A (LAMP2A) is a key regulator of chaperone-mediated autophagy. **(A)** Four positively charged amino acids (KHHH) in the C-terminal of LAMP2A are necessary to selectively bind with the negatively charged residues of the cytosolic cargo protein. **(B)** Under physiological conditions, μ-calpain does not have access to the luminal domain of LAMP2A, but activated μ-calpain (calpain 1) can access and cleave LAMP2A when LAMP2A multimerizes, forming a pore during chaperone-mediated autophagy. **(A, B)** Reprinted with permission from Rodriguez and Torriglia (2013). **(C)** Hsp70.1 contains two functional domains: N-terminal nucleotide-binding domain (NBD) and C-terminal substrate-binding domain (SBD). After separating from the NBD, the SBD loaded with the cargo protein passes through the LAMP2A multimeric tunnel. Adapted with permission from Dong et al. (2023).

Autophagy is an evolutionarily conserved cellular process through which parts of the cell are degraded within the lysosomes. It is classified into three processes, namely, macroautophagy, microautophagy, and chaperone-mediated autophagy (Figure 1A), through which intracellular damaged/aged macromolecules and organelles are degraded into recycle amino acids (Cuervo, 2004; Mizushima et al., 2008; Levine et al., 2011; Kaushik and Cuervo, 2018). To achieve each type of autophagy, the function of lysosomes is critical for maintaining homeostasis and protecting cells against stress. Initially, the best-characterized form of autophagy is a process of bulk trapping of the cytoplasm or damaged/aged organelles, which was designated as macroautophagy (De Duve, 1963). Double membrane-bound cytosolic cargo named autophagosome fuses with lysosomes, which provide hydrolytic enzymes for the degradation of the trapped cargo proteins, macromolecules, and organelles (Figure 1A). For the selective bulk degradation or assisting of chaperone-mediated autophagy, microautophagy entraps cytosolic cargo such as soluble or membrane-bound material or naïve Hsp70.1 in small vesicles formed by invagination at the lysosomal limiting membrane (Figure 1A–c). Although macroautophagy and microautophagy are observed even in simple model organisms such as yeast, worms, or flies, chaperone-mediated autophagy (Figures 1A-b, 2) is found only in birds and animals (Eskelinen et al., 2005).

Chaperone-mediated autophagy (Figures 1A-b, 2) not only regulates multiple physiological processes but also contributes to a variety of disease processes. For example, proteostasis, cellular energetics, and immune responses are all regulated by this type of autophagy. Its genetic blockage in the mouse liver revealed that chaperone-mediated autophagy plays a key role in the regulation of glucose and lipid metabolism (Schneider et al., 2014; Schneider et al., 2015). A decrease in chaperone-mediated autophagy makes cells susceptible to oxidative stress (Dice, 2007). Especially under stress conditions, chaperone-meditated autophagy facilitates the selective degradation of oxidized proteins (Cuervo and Dice, 1996; Arias and Cuervo, 2011). Chaperone-mediated autophagy is responsible for the degradation of ∼30% of cytosolic proteins under prolonged nutrient deprivation (Dice, 2007). Downregulation of chaperone-mediated autophagy is thought to occur physiologically with aging (Auzmendi-Iriarte and Matheu, 2021), and this decrease is linked with the occurrence of age-related disorders such as neurodegenerative diseases and cancers (Cuervo et al., 2004; Kon et al., 2011; Kaushik and Cuervo, 2018; Auzmendi-Iriarte and Matheu, 2021; Gómez-Sintes and Arias, 2021).

**FIGURE 3:**
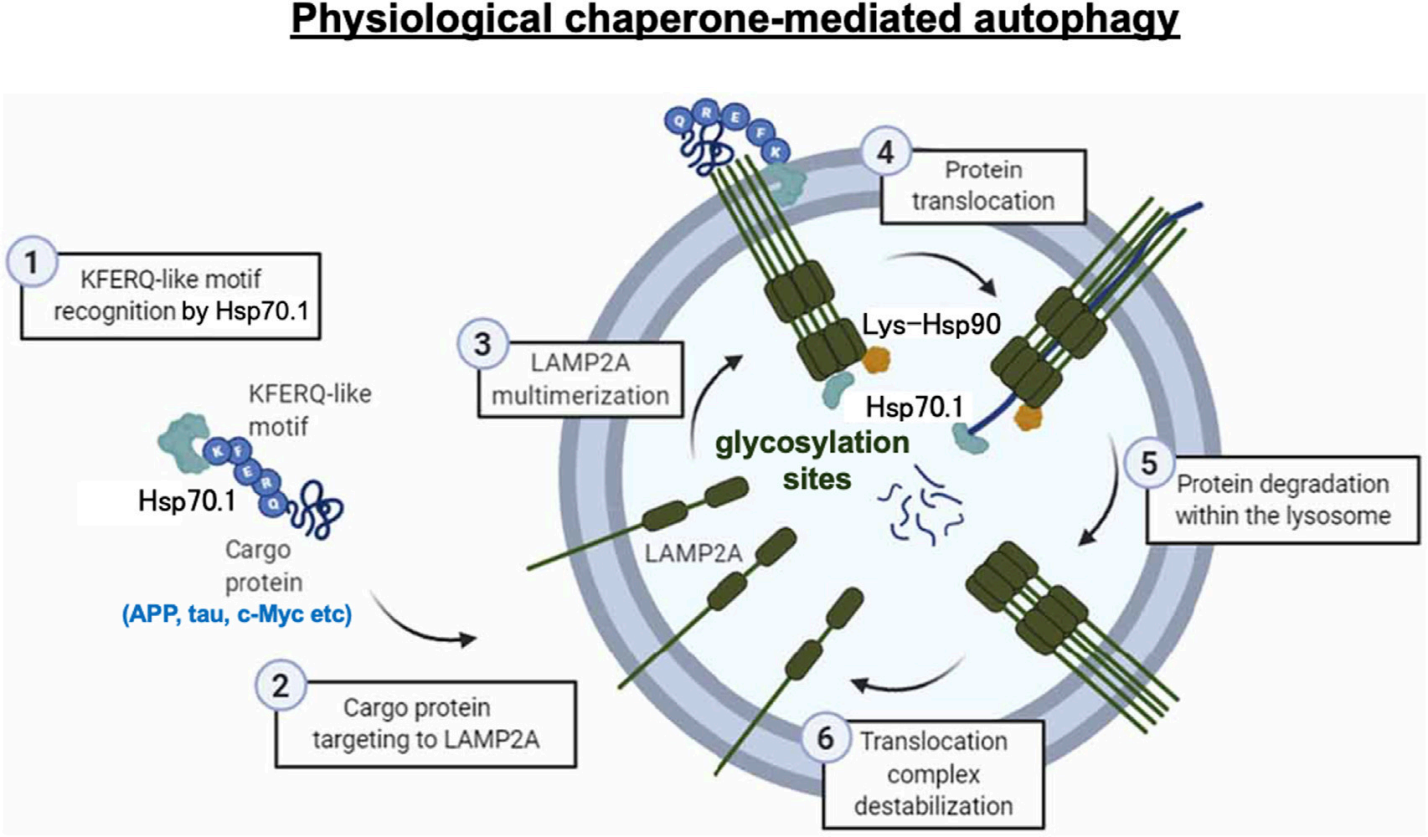
Chaperone-mediated autophagy under physiological conditions. Step 1: recognition of the KFERQ motif by Hsp70.1 (3-①). Step 2: targeting the Hsp70.1–cargo protein to LAMP2A (3-②). Step 3: multimerization of LAMP2A (3-③). Step 4: unfolding of the cargo protein and passage of the Hsp70.1–cargo complex through the LAMP2A multimer at the lysosomal limiting membrane (3-④). Step 5: cargo degradation (3-⑤). Step 6: LAMP2A multimer destabilization (3-⑥). As the APP, tau protein, c-Myc, etc., are representative proteins with the KFERQ motif, failure of chaperone-mediated autophagy, e.g., in Alzheimer’s disease, results in the accumulation of amyloid β and tau proteins. Reprinted with permission from Auzmendi-Iriarte and Matheu (2021).

Chaperone-mediated autophagy exerts the degradation of limited substrate proteins with a Lys-Phe-Glu-Arg-Gln (KFERQ) motif (Figures 1A-b, 2B, 3-①), which are delivered to the lysosomes by Hsp70.1 and co-chaperones such as Hsp40 (Dice, 1982; 1990). The KFERQ motif comprises a sequence of amino acids with specific charge and hydrophobicity. Approximately 30% of cell proteins containing the specific motif can cross the lysosomal limiting membrane to be degraded within lysosomes. For example, amyloid precursor protein (APP), tau protein, α-synuclein, c-Myc, TP53, and HIF-1α, which are believed to be central in the pathogenesis of neurodegenerative diseases and cancers, contain KFERQ motifs and are degraded by chaperone-mediated autophagy (Vogiatzi, et al., 2008; Wang et al., 2009). Accordingly, disorders of chaperone-mediated autophagy caused by Hsp70.1 dysfunction induce the accumulation of APP, tau protein, α-synuclein, etc. (Park et al., 2016; Robert et al., 2019). The KFERQ motif binding to Hsp70.1 brings the cargo protein to the lysosomal surface for docking, induces the multimerization of lysosome-associated membrane protein type 2A (LAMP2A), and enables the internalization of Hsp70.1 complexed with the cargo protein into lysosomes through the multimerized LAMP2A (Figures 2B, 3-②, ③) (Bandyopadhyay et al., 2008; Kaushik and Cuervo, 2018; Terasawa et al., 2021).

**FIGURE 4:**
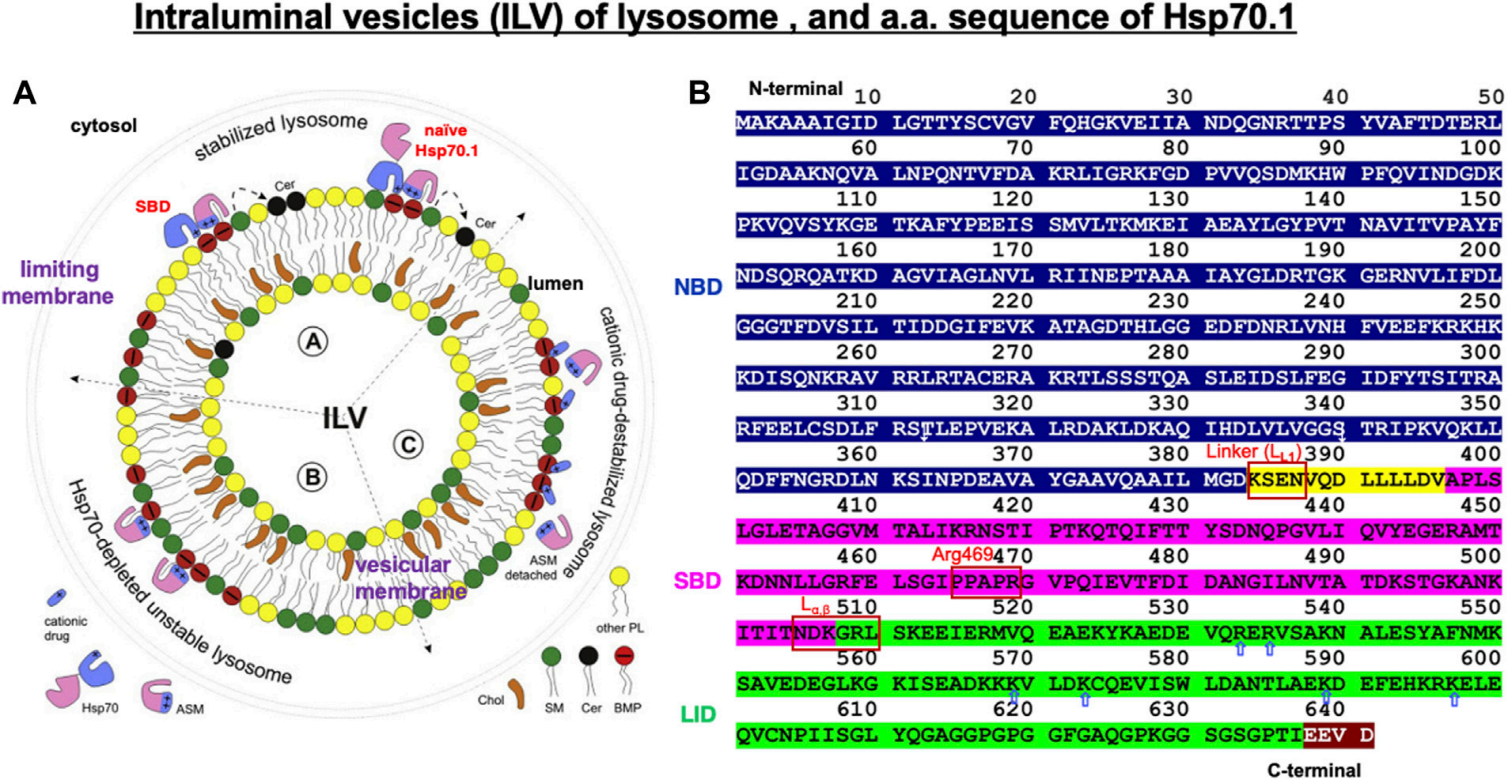
Structure of the intraluminal vesicle in lysosomes and the amino acid sequence of human Hsp70.1 domains. **(A)** Hsp70.1-mediated stabilization of the integrity of the lysosomal limiting membrane and intraluminal vesicle (ILV). ASM: acid sphingomyelinase; SM: sphingomyelin (green); Cer: ceramide (black); BMP: bis(monoacylglycero)phosphate (red). Adapted with permission from Balogi et al. (2019). **(B)** Domains in human Hsp70.1: the ATP/ADP-binding domain (1–383: NBD in blue), the flexible linker L_L1_ (384–396: yellow), the substrate-binding domain (397–507: SBD in magenta), the lid (508–641: green), and the C-terminal EEVD motif (638–641: brown). L_L1_, Arg469, and L_α,β_ are probable cleavage sites by activated μ-calpain. Blue, open arrows indicate positively charged residues in the lid of the SBD, which enables anchoring of the lid with negatively charged phosphatidylcholine of the cell membrane. The representative linker sites are indicated by the red rectangles. Adapted with permission from Vostakolaei et al. (2021).

When Hsp70.1 binding with cargo proteins docks at the lysosomal limiting membrane, the N-terminal, nucleotide-binding domain (NBD), of Hsp70.1 is not required for the membrane passage. Therefore, cleavage of the NBD from the SBD by activated μ-calpain may assist in the delivery of the smaller Hsp70.1–cargo complex through the LAMP2A multimer (Figure 2C). After the SBD–cargo protein complex is internalized, the SBD of Hsp70.1 dissociates from the cargo protein in the lysosomal lumen. Subsequently, the cargo-free SBD binds with bis(monoacylglycero)phosphate (BMP) (Figures 1B, 3, 4A) at the internal vesicular membranes, while the cargo protein is recycled into amino acids within the lysosomal lumen. It is likely that the separation of the SBD–cargo complex from the NBD and the unfolding of the substrate protein synergically facilitate their passage through the LAMP2A multimer into the lysosomal lumen (Figure 2C). However, the support of the full-length Hsp70.1 is necessary within the lysosomal lumen to pull the substrate protein into the LAMP2A multimer from the luminal side (Figure 3-④). For this purpose, uptake of the cytoplasmic naïve Hsp70.1 into late endosomes (Figures 1A-c) might have occurred via microautophagy prior to the lysosome–endosome fusion (Kaushik and Cuervo, 2018; Balogi et al., 2019).

## General scheme of chaperone-mediated autophagy

The lysosomal limiting membrane contains abundant glycosylated membrane proteins such as LAMP1, LAMP2, LIMP1/CD63, and LIMP2, which may form a continuous carbohydrate layer at the luminal leaflet to protect the membrane from degradation by lysosomal hydrolases (Fukuda, 1991; Li et al., 2016; Yamashima, 2023b). Abundant glycosylation in the large intraluminal domain of LAMP2 plays an important protective role against lysosomal hydrolases (Figures 2, 3). LAMP2A is one of the three splicing variants of the Lamp2 gene, which contains a cytosolic tail (Figure 2A) that differs from that of the other LAMP2 isoforms (Cuervo and Dice, 2000). Under stress conditions, LAMP2A plays a crucial role in the selective uptake of substrate proteins for chaperone-mediated autophagy (Cuervo and Dice, 1996). The cytoplasmic tail of the 12 amino acids (GLKHHHAGYEQF) of LAMP2A is required for the docking of the Hsp70.1–cargo complex at the lysosomal limiting membrane (Figure 2A) (Cuervo and Dice, 1996; 2000; Ikami et al., 2022). As the first step of chaperone-mediated autophagy, four positively charged amino acids (Figure 2A; KHHH) in the cytosolic tail of LAMP2A selectively bind with the negatively charged residues of the cytosolic cargo proteins (Figures 2A, B, 3-①, ②) (Cuervo and Dice, 2000).

**FIGURE 5:**
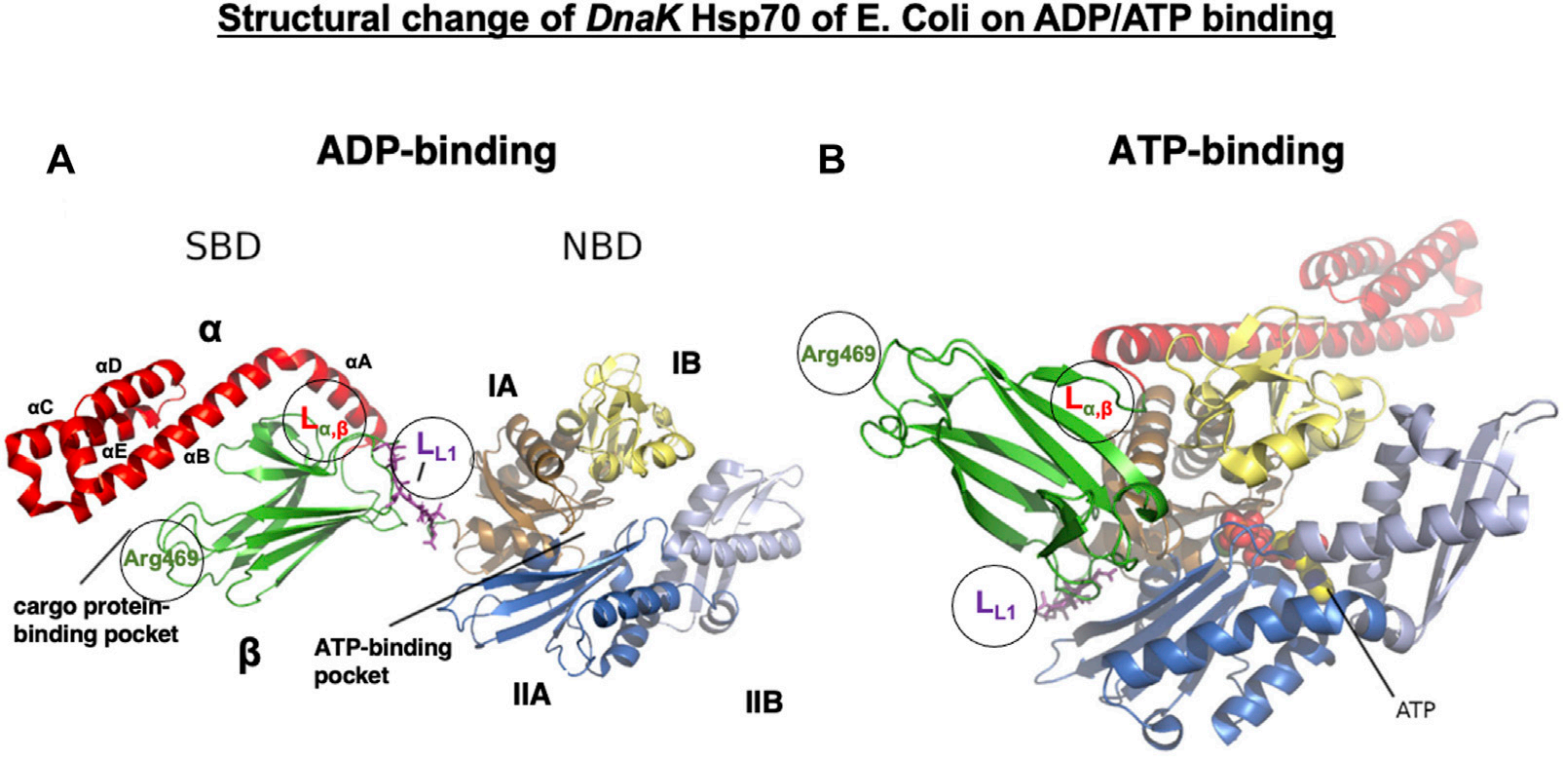
Conformational change in *E. coli Dna*K Hsp70 on ADP/ATP binding. *DnaK* Hsp70 consists of two domains of the C-terminal SBD and the N-terminal NBD, which are connected by a flexible and highly conserved hydrophobic linker (L_L1_, magenta sticks). The hydrolysis of ATP to ADP promotes NBD conformational changes, which are transduced through the L_L1_ linker to the SBD. The SBD consists of an eight-stranded β-sandwich subdomain (SBDβ, green) and an α-helical subdomain (SBDα, red) that docks onto the SBDβ like a lid. SBDβ and SBDα are connected by the linker (L_α,β_), which serves as a dynamic hinge appropriately positioning αB of the SBDα. In the ADP-bound state, SBDβ is tightly covered by SBDα **(A)**, but on ATP binding, SBDα is held open by the L_L1_ linker interaction with the ATPase region of the NBD, which is localized at the crevice between IA and IIA **(B)**. Note the translocation of the L_L1_ linker to the crevice on ATP binding. The NBD is formed of four subdomains: IA (tan), IB (pale yellow), IIA (marine blue), and IIB (light blue). They are organized into two lobes separated by a deep cleft, at the bottom of which ATP binds (B, spheres). The linker L_L1_, L_α,β_, and Arg469 all play roles in the conformational changes of Hsp70.1. Based on the molecular weight of the cleaved Hsp70.1 bands (Figure 7), L_L1_, L_α,β_, and Arg469 may be possible cleavage sites (circles) by activated μ-calpain. Adapted with permission from Havalová et al. (2021).

**FIGURE 6:**
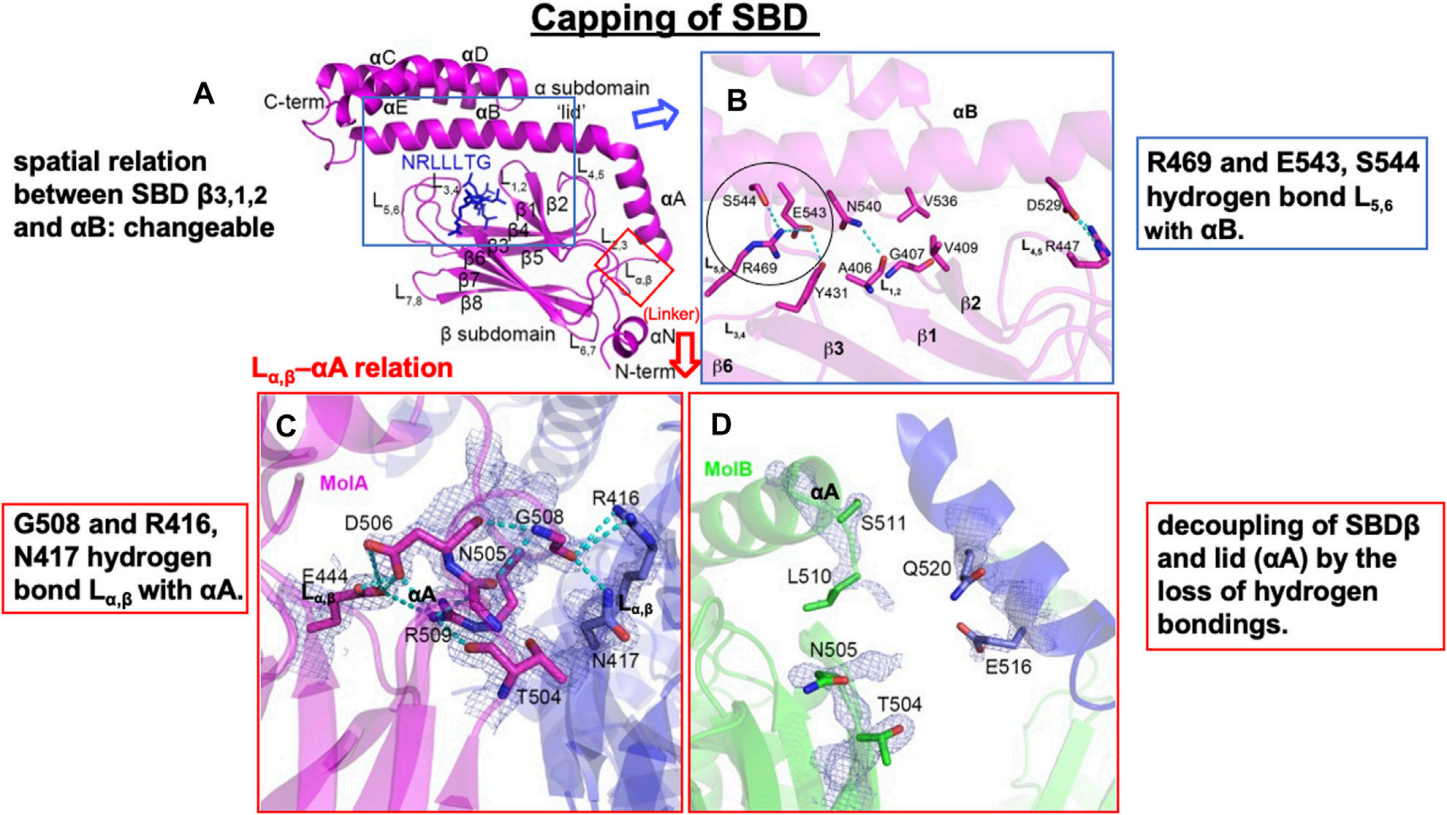
Flexible spatial relation between SBDβ and SBDα of human Hsp70.1. **(A)** SBD and certain client peptide NRLLLTG are shown in magenta and blue, respectively. **(B)** Close-up view of the interaction between the SBDαB and SBDβ subdomains of Hsp70.1. Irreversible conformational change may occur through carbonylation and the resultant cleavage of Arg469 (circle). **(C,D)** The L_α,β_–αA interaction is flexible, depending on the distinct protein cargo: interacting “molecule A (MolA)” and potential interacting “molecule B (MolB).” For example, “MolA” shows tight hydrogen bonding, whereas “MolB” shows decoupling of SBDβ from the lid (αA). It is likely that this flexible L_α,β_–αA relation facilitates the access of 4-hydroxy-2-nonenal (4-HNE) or activated μ-calpain. Hydrogen bonds are indicated with dotted lines. Blue rectangle in **(A)** is magnified in **(B)**, while the red rectangle in **(A)** is magnified in **(C,D)**. Adapted with permission from Zhang et al. (2014).

As a second step, after the cargo proteins interact with the cytoplasmic tail of LAMP2A, Hsp70.1 induces its multimerization (Figures 2B, 3-③). Multimerization of LAMP2A into a 700-kDa protein complex enables the influx of the SBD–cargo complex from the cytosol into the lysosomal lumen (Bandyopadhyay et al., 2008; 2010; Kaushik and Cuervo, 2018; Terasawa et al., 2021). Both of the above steps are indispensable for the selective uptake of the SBD–cargo complex into the lysosomal lumen. For the docking at the lysosomal surface, unfolding of the substrate protein is not required, but it is necessary for its passage through the LAMP2A multimer (Figure 3-④) (Salvador et al., 2000). As LAMP2A is the main effector of chaperone-mediated autophagy, its expression, trafficking, and stabilization are tightly regulated for the maintenance of cell and organism homeostasis. When LAMP2A monomers are assembled into multimeric structures (Figure 3-③), as part of step 3, the SBD–cargo complex passes through the tunnel of the LAMP2A multimer (Figures 2B, C, 3-③-④). During passage, naïve Hsp70.1 within the lysosomal lumen pulls the unfolded cargo protein, thus preventing its return back to the cytosol (Figure 3-④). Lysosomal Hsp90 helps stabilize LAMP2A by masking it from degradation by lysosomal protease-binding sites. Finally, the cargo protein is degraded into amino acids within the lysosomal lumen (Figure 3-⑤), and the multimerized LAMP2A complex is disassembled (Figure 3-⑥). Chaperone-mediated autophagy is impaired under stress conditions due to both changes in the LAMP2A level at the lysosomal membrane (Cuervo and Dice, 2000) and proteolytic degradation of the carbonylated Hsp70.1 in the cytosol (Yamashima et al., 2023a).

As discussed earlier, under pathological conditions or experimental stress conditions, e.g., in the cultured hepatoma cells of humans and the brain, liver, or pancreas tissues of monkeys, excessive activation of μ-calpain cleaves Hsp70.1 (Sahara and Yamashima, 2010; Yamashima et al., 2020; Boontem and Yamashima, 2021; Seike et al., 2022; Yamashima et al., 2023a; Yamashima, 2023b; Yamashima et al., 2023c; Yamashima et al., 2023d). In addition, in light-induced retinal degeneration and photoreceptor cell death, activated μ-calpain cleaves LAMP2A (Figure 2B) and induces lysosomal membrane permeabilization (Rodriguez and Torriglia, 2013). Because of the inducing failure of chaperone-mediated autophagy, the simultaneous cleavage of Hsp70.1 and LAMP2A by excessive μ-calpain activation may cause lysosomal disintegration (Miyazaki, 2023). We speculate that Hsp70.1 cleavage at or around the linker (L_L1_) bridging the NBD and SBD occurs through the physiological μ-calpain activation to facilitate the SBD–cargo passage through the LAMP2A multimer (Figures 2C, 4B, 5). However, under excessive μ-calpain activation during cell stress, the SBD may be unexpectedly cleaved in the cytosol before the Hsp70.1–cargo complex reaches the LAMP2A multimer. Furthermore, if the activated μ-calpain also cleaves LAMP2A (Rodriguez and Torriglia, 2013), the impairment of both chaperone-mediated autophagy and lysosomal membrane destabilization will occur.

## Hsp70.1-dependent lysosomal membrane integrity

Lysosomal membranes comprise both the limiting membrane, which forms a boundary with the cytosol, and the intraluminal vesicular membranes (Figures 1B, 4A). Full-length, naïve Hsp70.1, a protein that lacks a lysosomal leader sequence, enters the lysosomal lumen perhaps *via* microautophagy or direct membrane crossing (Balogi et al., 2019). A cluster of positively charged Arg and Lys residues (R533 to K597; Figure 4B, blue open arrows), which anchor Hsp70.1 to the endosomal/lysosomal membrane, enables its entry via microautophagy (Morozova et al., 2016). Negatively charged phosphatidylserine on the cytosolic leaflet of the endosomal/lysosomal membrane recruits the positively charged Hsp70.1 (Yeung et al., 2008), and Hsp70.1 oligomers generate pores in the membrane (Arispe et al., 2004; Lamprecht et al., 2018).

Lysosomes are cytosolic vesicles that recycle damaged/aged/misfolded proteins into amino acids, where ∼60 acid hydrolases (cathepsins being the most abundant) digest macromolecules of the cell for amino acid reutilization (Saftig and Klumperman, 2009; Settembre et al., 2013). The SBD of Hsp70.1 after separating from the cargo and/or the full-length naïve Hsp70.1 (Figure 4A) anchors to acid sphingomyelinase (ASM) at the intraluminal vesicular membranes to stabilize the lysosomes (Nylandsted et al., 2004; Kirkegaard et al., 2010). Both Arg (R) and Lys (K) residues of the SBD and positively charged domains of ASM serve as docking sites with the negatively charged head group of BMP at the intraluminal vesicular membranes (Figure 4A) (Balogi et al., 2019). The high-affinity association of the SBD/Hsp70.1 with BMP facilitates the binding of BMP to ASM, and this activates ASM (Figure 4A) (Kirkegaard et al., 2010, 2016).

Hsp70.1-dependent activation of ASM converts sphingomyelin to ceramide at the intraluminal vesicular membranes (Figure 4A), and such changes in the lipid composition contribute to the stabilization of lysosomal limiting membranes (Balogi et al., 2019). The mechanism by which ceramide stabilizes the limiting membranes remains largely unknown. While the presence of short–long-chain ceramides at the plasma membranes is thought to be a mediator of cell death, very-long-chain ceramide species (C24:0, C24:1, and C24:2) reinforce membrane integrity (Hartmann et al., 2012; Stiban and Perera, 2015; Rudd and Devaraj, 2018). An increased level of very-long ceramides at the limiting membrane counteracts the aggregation of lysosomes with other intracytoplasmic vesicles and membranes, which may strengthen limiting membranes from the cytoplasmic side (Heinrich et al., 2000). Simultaneously, the aggregation of numerous intraluminal vesicles may strengthen the limiting membrane from the luminal side.

Inside the lysosomes, iron and other metals can generate ROS via Fenton-type chemical reactions, which cause the oxidization and destabilization of membrane lipids (Kurz et al., 2010; Kiselyov et al., 2011). Localization of either the SBD or naïve Hsp70.1 in the lysosomal lumen effectively protects lysosomal membranes and inhibits their destabilization during local oxidative stress (Kirkegaard et al., 2010). Therefore, disorder of the SBD and depletion of naïve Hsp70.1 cause lysosomal membrane rupture or permeabilization. Cationic lysosomotropic drugs can neutralize the negative charge of BMP to which ASM and SBD/Hsp70.1 are anchored, thus inhibiting ASM function (Figure 4A) (Kölzer et al., 2004) and causing lysosomal instability. Since the key site Arg469 of Hsp70.1 is prone to specific oxidation injury (Oikawa et al., 2009), Hsp70.1 becomes vulnerable to calpain-mediated cleavage, especially after the Arg469 carbonylation (Yamashima and Oikawa, 2009). The resultant loss of lysosomal membrane integrity and the release of lysosomal hydrolases such as cathepsins into the cytosol are lethal to the cells.

## Role of L_L1_ in the *DnaK* Hsp70 conformational change in ADP/ATP binding

The Hsp70 family was first identified more than 50 years ago in *Drosophila* as 70-kDa proteins that are induced by heat stress (Ritossa, 1962; Schedl et al., 1978; Ashburner and Bonner, 1979). The Hsp70 family was considered the most conserved in evolution; it is present from *Escherichia coli* to humans. *DnaK* is the major bacterial Hsp70 found in the *E. coli* cytosol, being one of the most abundant, constitutively expressed, and stress-inducible chaperones. As plants must anticipate the upcoming severe temperature in summer days, overexpression of Hsp70 can improve their basal thermotolerance (Bourgine and Guihur, 2021). For human Hsp70.1, very little is known about the conformational change in the SBDβ and SBDα in complex with a client peptide (Zhang et al., 2014). So, much of our knowledge about Hsp70.1 structural changes is from analyses of *DnaK* Hsp70 (Figure 5), which is a member of the Hsc70 molecular chaperone family of *E. coli* (Suppini et al., 2004; Havalová et al., 2021). In both *DnaK* Hsp70 and human Hsp70.1, the C-terminal SBD and the N-terminal NBD are connected by a highly conserved interdomain linker L_L1_ (Figures 4B, 5), which modulates their allosteric rearrangement (Vogel et al., 2006; Swain et al., 2007; Kityk et al., 2012; Zhuravleva et al., 2012; Qi et al., 2013; Eugenia et al., 2019).

A crystal structure of the SBD shows SBDβ as a β-sandwich that contains the cargo protein-binding pocket, while SBDα is an α-helical subdomain that functions as a lid, covering the cleft of SBDβ (Figures 5, 6A) (Zhu et al., 1996; Wang et al., 1998). SBDα comprises five α-helices (αA, αB, αC, αD, and αE) (Figures 5, 6A). The SBDβ and SBDα subdomains are connected by the loop L_α,β_, which is less conserved among the species relative to the SBD (Figures 4B, 5). When binding with the client peptide under physiological conditions, conformational changes may occur in both L_α,β_ and L_L1_. The NBD comprises two lobes (I and II) separated by a central ATP/ADP-binding pocket, and each lobe is subdivided into two subdomains (IA and IB while IIA and IIB, respectively) (Figure 5) (Flaherty et al., 1990, 1991). Hsp70 exerts its functions by cycling between states of low and high affinity for the client polypeptide in a sequential manner, which is driven by ATP binding and hydrolysis and ADP–ATP exchange at the NBD. When ADP is bound to the NBD, which exhibits modest ATPase activity, the cargo protein binds with the SBD with high affinity (Figure 5A), whereas when ATP is bound, they interact weakly (Figure 5B).

On ADP binding, the cargo protein-binding pocket is covered by the α-helical lid, and the cargo protein is held tightly. When a conformational change occurs in ATP binding, it opens the α-helical lid of the SBD, the cargo protein-binding pocket in the SBD is uncovered, and the affinity of Hsp70 for the cargo protein is reduced (Arakawa et al., 2011). On ADP binding, the NBD and SBD subdomains tumble independently, tethered to each other only by the linker L_L1_ (Figure 5A). In contrast, on ATP binding, the NBD lobes rotate relative to each other, SBDα and SBDβ subdomains dock to different faces of the NBD detaching from each other, and the L_L1_ linker binds into a crevice between NBD IA and IIA (Figure 5B). Accordingly, when Hsp70 releases the ADP and binds another ATP molecule, it induces substantial structural rearrangements of domains (Figure 5B). This is the reason why the signal of ATP binding in the NBD can be immediately transmitted through the flexible tether L_L1_ to the SBD. Such a conformational change exerts force onto the folded client protein to unfold via direct interactions of Hsp70 (Rohland et al., 2022).

## Role of L_α,β_ and Arg469 in human Hsp70.1 conformational change under stress conditions

In ATP-bound *DnaK* Hsp70, L_α,β_ serves as a dynamic hinge that serves to appropriately position the long helix αB of SBDα in close vicinity of the NBD (Figure 5B) (Kityk et al., 2012; Qi et al., 2013; Zhang et al., 2014). A sequence alignment of this region reveals significant differences between *DnaK* Hsp70 and human Hsp70.1. In Hsp70.1, L_α,β_ is composed of conserved amino acids with larger side chains (NDKGRL) (Figure 4B). The binding pocket of a substrate peptide (e.g., “NRLLLTG”) is localized between L_3,4_ and L_1,2_, and the binding cleft is further stabilized by L_5,6_ and L_4,5_ through a series of hydrogen bonds and multiple van der Waals interactions between hydrophobic residues (Figures 6A, B) (Zhang et al., 2014).

Human-type Hsp70.1 traffics nascent or misfolded peptide substrates from the cytoplasm into the lysosomes for degradation. In Hsp70.1, the interactions in the C-terminal part of helix αB with L_3,4_ and L_5,6_ are flexible (Figures 6A, B). For example, Arg(R)469 and Glu(E)543/Ser(S)544 form hydrogen bonds between L_5,6_ and αB. Since Arg469 plays a key role in the interaction between αB of SBDα and the loops of SBDβ (Figure 6B, circle), an irreversible conformational change in Hsp70.1 may occur by the oxidation of Arg469. The other interactions between αB of SBDα and the loops of SBDβ also serve to rigidly link αB and the preceding αA to SBDβ, but they leave the C-terminal part of αB and the associated helical bundle region of the SBDα subdomain (αC and αD) to take on more divergent positions (Figures 5, 6A) (Zhang et al., 2014). The actual binding mode of the pocket and the lid may depend on the composition of the cargo protein and the microenvironment, i.e., the extent of cell stress. We speculate that the flexible conformational change in αB of SBDα and the loops of SBDβ during cell stress, i.e., the change in the spatial relation between the substrate-binding pocket and the lid, is closely related to whether activated μ-calpain can access the key sites of Hsp70.1 (Figure 5). It is likely that the conformational change in the SBDβ–αB spatial relation may occur when the key site Arg469 is carbonylated by 4-HNE and cleaved by activated μ-calpain (Figures 5, 6B).

In *E. coli DnaK* Hsp70, L_α,β_ is composed of residues with small side chains (ASSGL or SSSGL), whereas in human Hsp70.1, it is composed of a larger side chain (NDKGRL) (Figure 4). The crystal structure of a synthetic client peptide NRLLLTG-bound Hsp70.1 SBD (Figure 6A) reveals two distinct but very similar molecules of the complex per asymmetric unit cell between “molecule A (MolA)” (Figure 6C) and “molecule B (MolB)” (Figure 6D). The dramatically different conformational change in the L_α,β_–αA relation occurs between “MolA” and “MolB” (Figures 6C, D). In “MolA,” the L_α,β_ linker is involved in crystal packing through interactions with a loop from an adjacent molecule. In particular, the carboxylate oxygen atom of Gly(G)508 of L_α,β_ hydrogen bonds with the Arg(R)416 and Asn(N)417 residues of αA, and these interactions hold L_α,β_ and αA in a fixed position (Figure 6C). On the contrary, in “MolB,” the corresponding linker region is not observed because the adjacent molecules are more than 5Å away, showing decoupling of SBDβ from αA (Figure 6D) (Zhang et al., 2014).

## Calpain-mediated cleavage of carbonylated Hsp70.1

**FIGURE 7:**
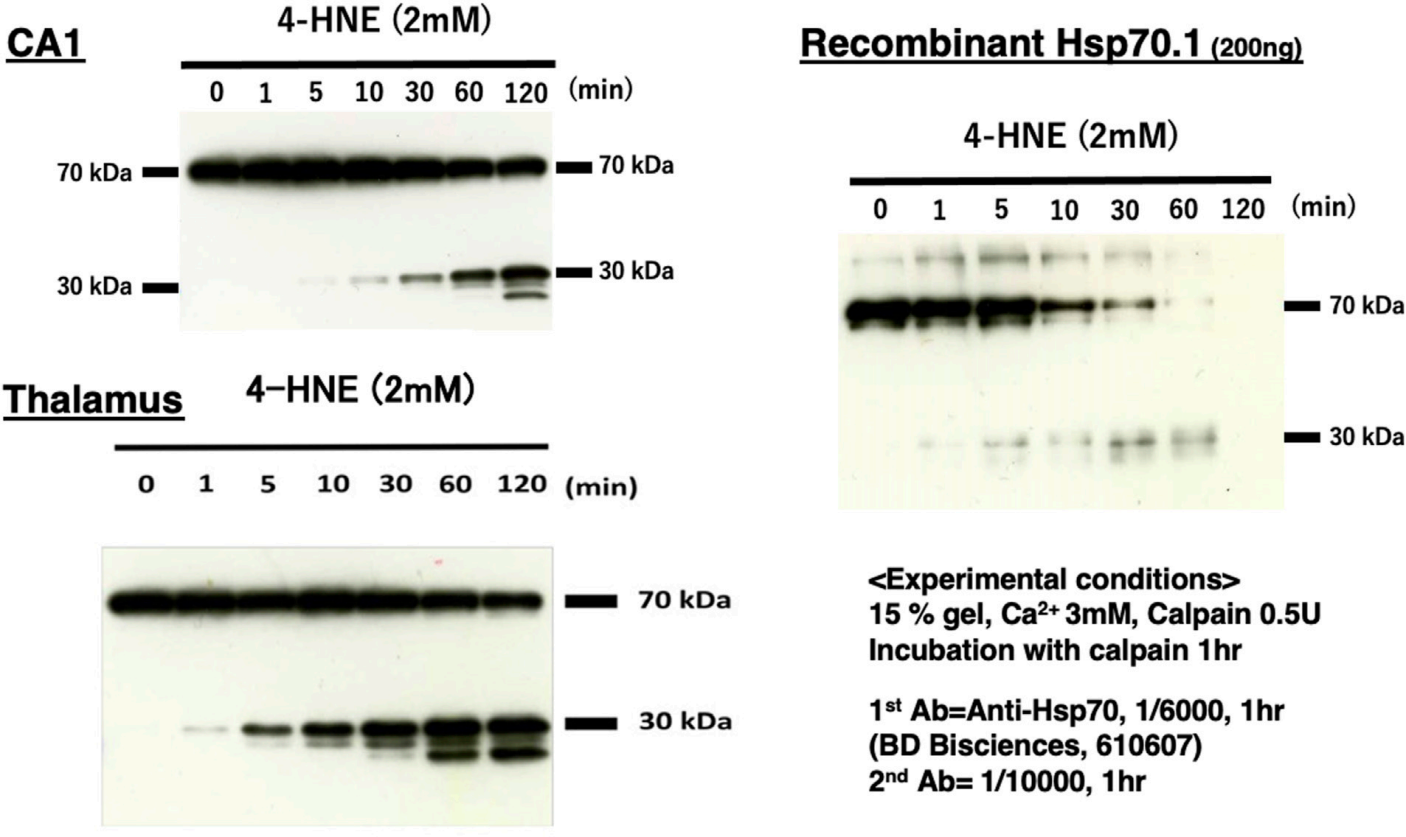
Calpain-mediated cleavage of carbonylated Hsp70.1. Under physiological conditions *in vivo*, the intracellular Ca^2+^ concentration is tightly regulated, being approximately 1–2 mM at the synaptic cleft, whereas it is ∼100 nM in the cytosol. Under pathological conditions, however, regulatory mechanisms are overwhelmed, and the intracellular Ca^2+^ concentration increases remarkably due to the influx from extracellular pools or release from endoplasmic reticulum stores. This is sufficient for activating μ-calpain (Yamashima, 2004). To activate μ-calpain *in vitro*, 3 mM of CaCl_2_ was added with 0.5 unit of crude μ-calpain to the monkey hippocampal CA1 and thalamus (10 μg) tissues and human recombinant Hsp70.1 (200 ng). They were incubated with 2 mM of synthetic 4-HNE for 1–120 min. For the Western blotting analysis, a mouse anti-human Hsp70.1 antibody (BD Biosciences, 610607) that recognizes amino acid 429–640 residues was utilized. In both brain-derived Hsp70.1 and the recombinant protein, the 4-HNE-mediated carbonylation of Hsp70.1 facilitated time-dependent cleavage by activated μ-calpain. The molecular weight of the cleaved bands of Hsp70.1 comprised the main band around 30 kDa and two additional bands around 20 kDa. Moreover, 4-HNE was thought to play a principal role in Hsp70.1 cleavage because activated μ-calpain alone could not cleave Hsp70.1 (4-HNE, 0 min) in this experimental paradigm. Reprinted with permission from Liang et al. (2016).

It is extremely difficult to detect the substrate of activated μ-calpain *in vivo* because calpain cleaves the substrate protein within seconds and without binding. Through the 2D electrophoresis assessment comparing the proteolysis of the monkey retina tissues under hypoxic conditions with or without calpain activation, Nakajima et al. (2006) found calpain breakdown of vimentin, β-tubulin, α-enolase, and Hsp70.1. We conducted *in vitro* cleavage assay using the monkey brain tissues and recombinant human Hsp70.1. This showed that Hsp70.1, being involved in the brain tissue, is prone to cleavage by activated μ-calpain, especially after oxidative modification by synthetic 4-HNE or hydrogen peroxide (Sahara and Yamashima, 2010; Liang et al., 2016). For example, similar results were obtained in the hippocampal CA1 and thalamus tissues of the monkey brain and recombinant human Hsp70.1, which indicated calpain-mediated cleavage of the carbonylated Hsp70.1 from 70 kDa to ∼30 kDa (Figure 7). As an anti-Hsp70.1 polyclonal antibody we utilized recognized amino acid 429–640 residues (most of the SBD) of human Hsp70.1, and 3 cleaved bands below 30 kDa were detected. However, in the absence of 4-HNE or hydrogen peroxide, activated μ-calpain alone could not cleave Hsp70.1 in both the brain tissues and the recombinant protein. It is likely that the oxidative stress-induced conformational change in the SBD helps activated μ-calpain gain access to the linker regions such as L_L1_, L_α,β_, or Arg469 of the SBD molecules (Figure 5). For the transmembrane passage of the Hsp70.1–cargo complex under physiological conditions, the NBD is no longer necessary once Hsp70.1 is docked at LAMP2A. Cutting down the NBD at the SBD–NBD linker L_L1_ may help the passage of the SBD–cargo complex through the LAMP2A multimer. Under pathological conditions, however, cleavage of the carbonylated Arg469 by activated μ-calpain would be increased.

The molecular weight of the cleaved bands of Hsp70.1 comprised the main band around 30 kDa and two additional bands around 20 kDa in both the brain tissues and recombinant Hsp70.1 after the 4-HNE treatment (Figure 7). The cleavage site of ∼30 kDa was thought to be around or within the linker L_L1_ that connects the NBD and SBD (Figures 4B, 5). If the cleavage site is Lys384 at the N-terminal of L_L1_, the molecular weight may be approximately 27.9 kDa. If 30 kDa is the precise size of the main band, the cleaved site may be around Lys361 in the IA of the NBD. Furthermore, when the carbonylated Arg469 or L_α,β_ was exposed to activated μ-calpain after the conformational change, the estimated size of the cleaved bands may be 18.7 or 15.0 kDa, respectively (Figure 4B).

Both the lysosomal membrane disintegrity and autophagy failure due to the Hsp70.1 disorder will contribute to the development of lysosomal cell death. The latter potentially has two opposite effects, i.e., one is detrimental for the occurrence of neurodegenerative diseases, while the other is beneficial for the treatment of cancers. If certain compounds can protect the cells from oxidative stress or selectively injure the key binding sites of Hsp70.1, including Arg469, L_α,β_, and L_L1_, it would be a significant medicine for treating either neurodegenerative diseases or cancers. Accordingly, further analyses are necessary to precisely determine the cleaved sites in Hsp70.1, especially under stress conditions, to help the development of compounds that can modify key sites of Hsp70.1 as a novel medicine for neurodegenerative diseases or cancers.

## Loss of Hsp70.1 function due to cleavage induces lysosomal membrane rupture

**FIGURE 8:**
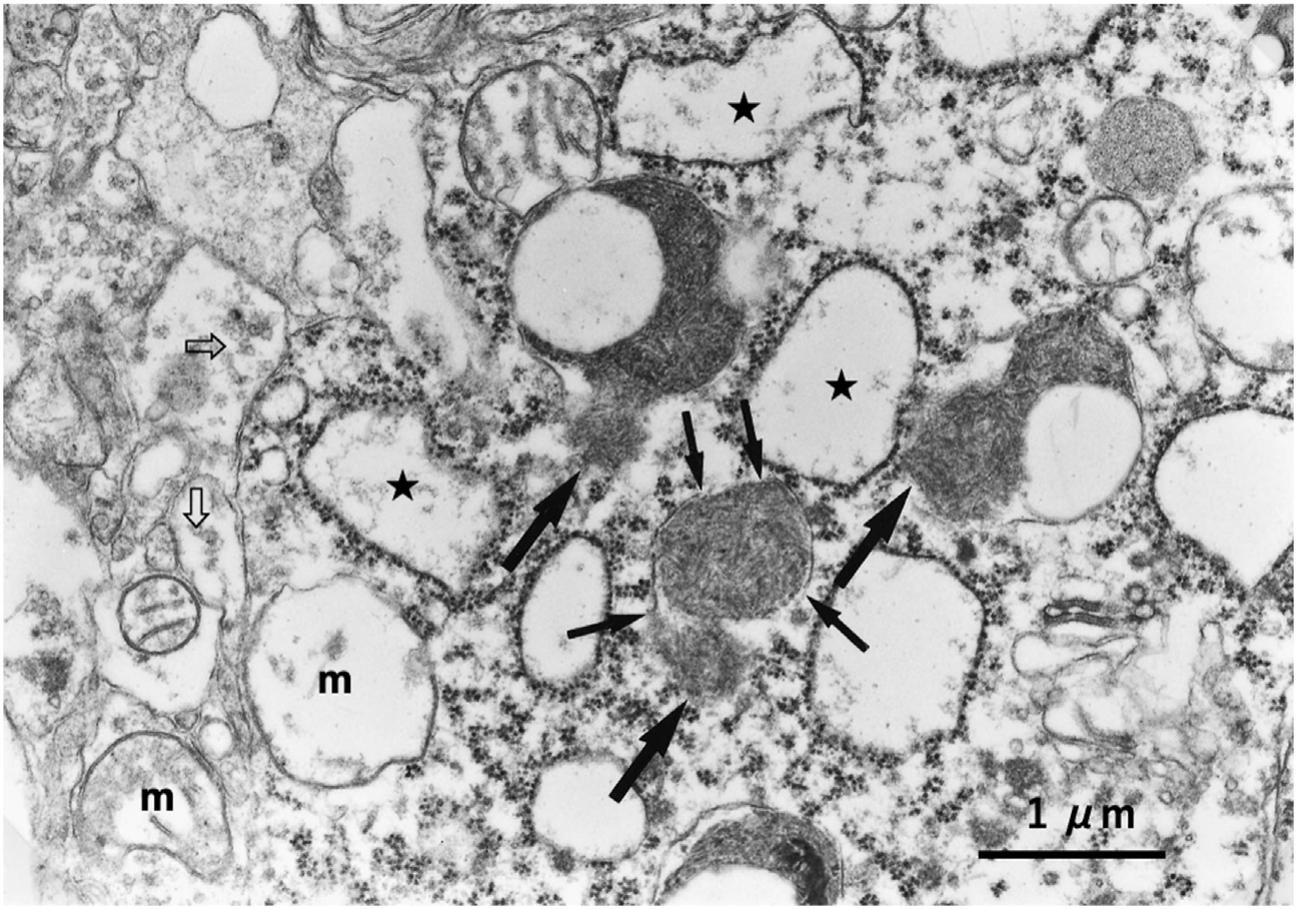
Ultrastructural evidence of lysosomal membrane rupture. Evidence of the lysosomal membrane rupture (small arrows) with the leakage of the lysosomal content (large arrows), which was found in the monkey hippocampal CA1 neuron after transient global brain ischemia. Stars indicate swollen rough ER, “m” indicates degenerated swollen mitochondria, and open arrows indicate a marked decrease in synaptic vesicles.

Recently, Yamashima (2023b) reported that the leakage of lysosomal cathepsins occurs either by the apparent disruption of the lysosomal membrane (rupture) and/or through the ultrastructurally blurred membrane (permeabilization) regardless of the insults, cell types, organs, diseases, or species in both experimental models and clinical materials. Since both lysosomal membrane rupture and permeabilization occur in the early phase of cell death, very few intact or even partially degenerating lysosomes would be detected by the ordinary electron microscopic analysis in both animal tissues and human disease samples. However, from cultured cells to human tissues, evidence of lysosomal membrane rupture was found by the careful electron microscopic analysis of consecutive ultrathin tissue sections (Figure 8) (Yamashima et al., 1996; Yamashima, 2023b).

Previously, we demonstrated that ω-6 PUFA induces the activation of μ-calpain via Ca^2+^ mobilization after binding with G protein-coupled receptors like GPR40 (Yamashima et al., 2020; Yamashima et al., 2023a; Yamashima, 2023b; Yamashima et al., 2023d), as well as GPR109A and GPR120 (Boontem and Yamashima, 2021; Seike et al., 2022). For example, Western blotting analysis of the pancreatic tissue after consecutive injections of synthetic 4-HNE in monkeys showed increases in μ-calpain activation and Hsp70.1 cleavage, which were associated with the expression of the 4-HNE receptor GPR109A (Boontem and Yamashima, 2021). In addition, Seike et al. (2022) confirmed the expression of GPR120 in both the human and monkey liver. Using HepG2-cultured hepatocytes exposed to 4-HNE, they demonstrated that the effects of 4-HNE are regulated by activated µ-calpain via GPR120. These data indicate that 4-HNE can activate GPR40, GPR109A, and GPR120 in neurons, β-cells, and hepatocytes, respectively, to induce Ca^2+^ mobilization, which is sufficient for μ-calpain activation.

Additionally, 4-HNE is generated by high-temperature frying of linoleic acid-rich vegetable oils (Yamashima et al., 2020; Boontem and Yamashima, 2021; Seike et al., 2022). The serum concentration of 4-HNE is determined not only by the long-term intake of deep-fried foods (exogenous) but also by the oxidation of biomembranes by circumferential oxidative stress for long years (endogenous) (Schaur et al., 2015; Yamashima et al., 2020). Moreover, 4-HNE is an amphiphilic molecule, water-soluble but with strong lipophilic characteristics, enabling its diffusion throughout the body organs (Zheng et al., 2014; Bekyarova et al., 2019). Aldehyde dehydrogenase 2 (ALDH2), an enzyme found in the mitochondrial matrix, detoxifies not only alcohol-derived acetaldehyde but also ω-6 PUFA-derived 4-HNE. Approximately 540 million people, i.e., 8% of the world population, have a remarkable reduction in ALDH2 activity due to a missense mutation in its gene (Chen et al., 2008; Seike et al., 2022). Furthermore, an age-dependent decrease in ALDH2 activity in all subjects after the age of 40 years has been described (Schaur et al., 2015). Therefore, hereditary-reduced or null ALDH2 activity, aging, and consumption of deep-fried foods (Yamashima et al., 2020) all contribute to increased 4-HNE accumulation. Increases in both endogenous and exogenous 4-HNE, combined with age-dependent ischemia of each organ, may activate µ-calpain, which will cleave the lysosomal stabilizer Hsp70.1, especially after its carbonylation, and induce lysosomal cell death *via* cathepsin leakage. We propose that 4-HNE from both exogenous and endogenous sources may contribute to various age-related pathologies, at least in part, by adduction onto Hsp70.1, which, in turn, causes impairment in chaperon-mediated autophagy and lysosomal rupture (Figures 1B, 4A).

## Concluding remarks

Hsp70.1 has dual functions as a lysosome stabilizer and as a chaperon-mediated autophagy mediator, but the role of Hsp70.1 in several human pathologies is underestimated. We suggest that 4-HNE adduction onto Hsp70.1 leads to impairment in these important functions for cell viability. The levels of 4-HNE increase in the serum and organs both through the intake of deep-fried foods cooked using linoleic acid-rich vegetable oils (exogenous) and oxidation of biomembranes by oxidative stress (endogenous), especially in those with ALDH2 reduction. Therefore, lowering 4-HNE levels by lifestyle changes, activation of 4-HNE metabolizing enzymes, and/or compounds that protect Hsp70.1 from 4-HNE carbonylation may all provide means to reduce the burden of many lifestyle-related diseases.

## Abbreviations

ASM, acid sphingomyelinase; ALDH2, aldehyde dehydrogenase 2; APP, amyloid precursor protein; BMP, bis(monoacylglycero)phosphate; Hsp, heat shock protein; Hsc70, heat shock cognate protein 70; Hsp70.1, heat shock protein 70.1; 4-HNE, 4-hydroxy-2-nonenal; LAMP2A, lysosomal membrane protein type 2A; NBD, nucleotide-binding domain; PUFA, polyunsaturated fatty acid; ROS, reactive oxygen species; SBD, substrate-binding domain.
